# Membrane protein orientation and refinement using a knowledge-based statistical potential

**DOI:** 10.1186/1471-2105-14-276

**Published:** 2013-09-18

**Authors:** Timothy Nugent, David T Jones

**Affiliations:** 1 Bioinformatics Group, Department of Computer Science, University College London, Gower Street, London WC1E 6BT, UK

**Keywords:** Membrane protein, Statistical potential, Orientation, Refinement, Genetic algorithm

## Abstract

**Background:**

Recent increases in the number of deposited membrane protein crystal structures necessitate the use of automated computational tools to position them within the lipid bilayer. Identifying the correct orientation allows us to study the complex relationship between sequence, structure and the lipid environment, which is otherwise challenging to investigate using experimental techniques due to the difficulty in crystallising membrane proteins embedded within intact membranes.

**Results:**

We have developed a knowledge-based membrane potential, calculated by the statistical analysis of transmembrane protein structures, coupled with a combination of genetic and direct search algorithms, and demonstrate its use in positioning proteins in membranes, refinement of membrane protein models and in decoy discrimination.

**Conclusions:**

Our method is able to quickly and accurately orientate both alpha-helical and beta-barrel membrane proteins within the lipid bilayer, showing closer agreement with experimentally determined values than existing approaches. We also demonstrate both consistent and significant refinement of membrane protein models and the effective discrimination between native and decoy structures. Source code is available under an open source license from http://bioinf.cs.ucl.ac.uk/downloads/memembed/.

## Background

Although transmembrane proteins are encoded by approximately 30% of a typical genome and play vital roles in a diverse range of essential biological processes, they constitute only about 2% of structures deposited into the Protein Data Bank (PDB) [1,2]. This paucity of structures has meant that the majority of computational tools developed to analyse transmembrane proteins have focused on topology prediction [3-7] and de novo structure prediction [8-14]. Recently however, the increase in the number of solved crystal structures has led to the development of a number of automated methods with which to systematically and objectively analyse transmembrane proteins.

Transmembrane proteins differ from globular proteins in that they are embedded in the anisotropic environment of the lipid bilayer, composed of a heterogeneous mixture of lipid types with a central hydrocarbon core and a steep polarity gradient. Their positioning within the membrane is crucial to their folding, stability and activity yet the difficulties associated with crystallising transmembrane proteins in intact membranes mean that experimental orientation data is extremely scarce. While manual assessment has in the past been used to orientate transmembrane proteins [15], such strategies are poorly suited to large scale positioning on a genome-scale, and therefore automated computational approaches are increasingly important. Current methods include coarse-grained molecular dynamics simulations which have been used for large-scale positioning using a semi-quantitative lipid model [16,17]. While simulations have been shown to successfully reproduce the behaviour of equivalent atomistic simulations and peptide insertion experiments, molecular dynamics simulations are invariably slow and computationally expensive. The PPM/OPM method uses an anisotropic solvent model of the lipid bilayer, with polarity profiles derived from electron paramagnetic resonance studies, in combination with a grid search to minimise transfer energy from water to the membrane, with results correlating well with experimentally determined tilt angles and membrane thickness [18-20]. The TMDET algorithm calculates the membrane-exposed water accessible surface area of the target structure, followed by an exhaustive orientational search using an objective function which measures the fitness of a given membrane position to the protein [21-23]. However, in the absence of comparison with experimental studies, the accuracy of the approach is difficult to ascertain. Ez-3D implements a knowledge-based potential generated from the distribution of residues at varying membrane depths in 76 alpha-helical and 35 beta-barrel proteins, again employing a grid search to identify the global energy minimum [24-26]. Results are comparable to OPM and enable the generation of complete pseudo-energy topological landscapes that underscores positional stability, although the method is slower with a computation time of approximately 1 second per 5 residues.

While commonly used in globular protein structure prediction, the use of statistical potentials derived from transmembrane proteins is comparatively rare due to the low number of high-resolution structures deposited in the PDB. In the absence of structural data, methods such as FILM [8] attempted to construct a statistical potential via the analysis of 640 transmembrane helices belonging to 133 transmembrane proteins extracted from SWISS-PROT [27] with experimentally defined topologies, allowing small transmembrane proteins to be folded to a reasonable level of accuracy when combined with standard FRAGFOLD energy terms [28]. Later, an implicit membrane potential developed using 46 alpha-helical transmembrane protein structures was tested on various proteins as well as single transmembrane helices, demonstrating that in most cases the correctly inserted conformation was found to be at a clear energy minimum. These results indicated that the use of transmembrane amino acid distributions to derive an implicit membrane representation yielded meaningful residue potentials [29]. More recently, a membrane-specific modification of Rosetta included a membrane environment term derived from the analysis of 28 structures, scoring conformations by maximising the exposure of surface hydrophobic residues within the membrane and minimising hydrophobic exposure outside of the membrane [12-14]. In combination with additional Rosetta potentials modified to model the effect of the membrane environment including solvation and hydrogen bond terms, several small transmembrane protein domains (<150 residues) could be modelled to near-atomic accuracy (<2.5 Å).

In this paper, we present a computational approach for orientating both alpha-helical and beta-barrel transmembrane proteins in the lipid bilayer, employing a knowledge-based potential developed using the largest data set of transmembrane protein crystal structures yet assembled for statistical analysis. By using a combination of genetic (GA) and direct search algorithms to efficiently optimise positioning, our method is able to quickly and accurately identify native tilt angles, with results showing closer agreement with experimentally determined values than existing methods. We also report the ability of the potential to guide structure prediction by demonstrating consistent improvement in transmembrane protein model refinement and the effective discrimination between native from decoy structures.

## Results

### Comparison with OPM

**Figure 1:**
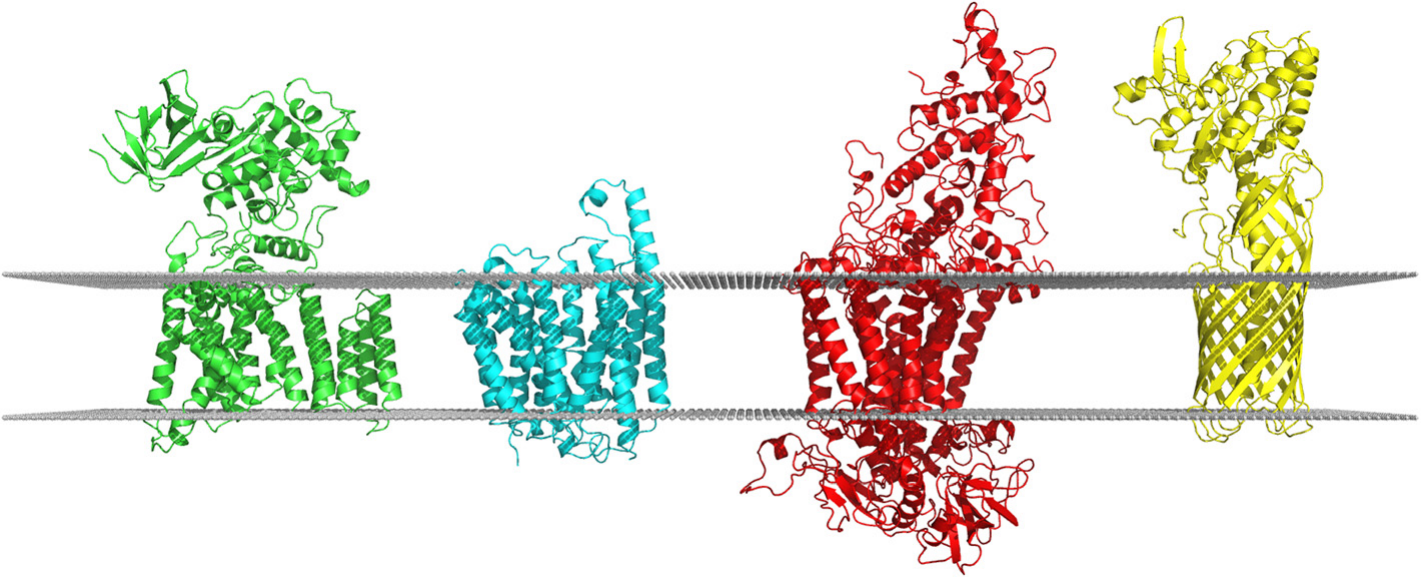
**Orientation of alpha-helical chains 3rceA, 3o7qA, 1dxrM and beta-barrel 3kvnA.** The grey lines, indicating the approximate position of the membrane, are placed at z = 15 Å and z = -15 Å.

Table 1 shows the cross-validated performance of a range of search strategies in positioning targets to within the published error margin of OPM. For GA searches, targets were positioned five times with the lowest energy orientation reported. The GA achieved best performance with 86.9% (159) of alpha-helical chains positioned to within the published error margin of OPM, with a mean tilt angle delta of only 1.07 degrees and a mean z-coordinate shift of 2.12 Å (Figure 1), suggesting good agreement with OPM. Using both direct and grid searches, results were similar although in the case of direct search the maximum observed tilt error was significantly larger (28.44 degrees compared to 7.61 degrees) indicating that local minima may have been encountered.

**Table 1:** Cross-validated results showing the performance of the three search strategies in positioning targets to within the published error margin of OPM

			**Within OPM error**		**Outside OPM error**		**All targets**	
**Search type**	**Type**	**Within OPM error**	**Mean tilt**	**Max tilt**	**Mean tilt**	**Max tilt**	**Mean tilt**	**Max tilt**
GA	Alpha	86.9%	0.56	2.98	3.58	7.61	1.07	2.12
Direct	Alpha	80.3%	0.77	4.83	6.94	28.44	1.98	2.66
Grid	Alpha	79.2%	0.74	3.74	5.04	14.68	1.63	1.67
GA	Beta	80.6%	1.03	3.62	9.25	20.02	3.77	3.82
Direct	Beta	83.3%	1.76	6.90	13.71	29.97	3.75	3.03
Grid	Beta	77.7%	2.35	10.03	14.12	37.64	4.97	2.74

Type indicates alpha-helical or beta-barrel. Tilt angles are the deltas compared to OPM and are measured in degrees. Mean z-shift is the mean z-coordinate delta compared to OPM calculated using all transmembrane segment boundary residues, measured in angstroms.

Despite the substantially lower number of structures used to generate the beta-barrel potential, 83.3% of targets were positioned to within the published error margin of OPM using direct search, reflecting the limited diversity of beta-barrel folds in contrast to alpha-helical structures. The mean z-coordinate shift of 3.03 Å indicates that beta-barrels are slightly harder to position along the z-axis although this could be a consequence of the larger translation per residue (~3.5 Å) in beta-strands compared to alpha helices (~1.5 Å) [30], suggesting that z-axis positioning of beta-barrels could benefit from a potential composed of thicker z-slices. Performance using the GA was similar (80.6%) with the maximum observed tilt error slightly lower at 20.02 degrees. For both alpha-helical and beta-barrel targets, results using a grid search were slightly worse than GA or direct searches, suggesting that the rotation and translation step size is preventing a lower energy from being found. We also tested a naïve approach which orientated structures by tilting them such that the longitudinal axis was parallel to the z-axis, and the mean z-coordinate set to z = 0. Only 2.2% of alpha-helical structures were correctly positioned to within the published error margin of OPM, with a large mean tilt angle delta of 15.7 degrees and a maximum error of 54.5 degrees, although the mean z-coordinate shift of 2.63 Å was more reasonable.

### Comparison with experimentally determined tilt angles

The use of detergents for membrane solubilisation during transmembrane protein crystallisation means that information regarding the positions of lipid molecules in crystallographic data is extremely rare, making comparison with experimentally determined tilt angles difficult. A small number of transmembrane proteins have had their tilt angles determined using Attenuated Total Reflectance Fourier Transform Infrared (ATR-FTIR) spectroscopy. We assessed performance of the potential, using GA search, and 4 other methods – OPM, TMDET, Ez-3D and a potential derived from experimental measurements of free energy of membrane insertion described by Hessa et al. [31] combined with a grid search - with these structures, comparing the mean absolute tilt angle of all transmembrane segments with the experimentally determined values (Table 2). Hessa et al. used systematically designed hydrophobic segments to quantitatively analyse the position-dependent contribution of all 20 amino acids to membrane insertion efficiency. Results show that in all cases, tilt angles calculated by our potential correlate well with ATR-FTIR values. Given the large experimental error, we suspect that all five methods produce more or less equivalent estimates. In most cases the experimentally determined values are systematically larger, possibly due to orientational disorder under experimental conditions, suggesting that these experimental values represent the upper limits of the actual tilt angles [18].

**Table 2:** Cross-validated results showing the performance of the potentials in positioning five targets with experimentally determined tilt angles

**Protein**	**PDB**	**Type**	**TM Subunits**	**MP tilt**	**OPM tilt**	**TMDET tilt**	**Ez-3D tilt**	**Hessa tilt**	**Experimental tilt**
Lactose permease	1 pv6	Alpha	1	24.9	22.1	22.7	22.1	22.2	33
FhuA	1qfg	Beta	1	40.0	38.8	39.2	-	-	46
OmpA	1qjp	Beta	1	39.6	38.3	38.5	-	-	44.5
KcsA channel	1r3j	Alpha	4	31.8	31.5	31.5	31.3	30.5	33
Phospholamban	1zll	Alpha	5	24.9	22.5	23.8	14.0	24.4	28 ± 6

Our potential is 'MP’. Tilt angles are the average of the absolute tilt angles of all transmembrane segments, measured in degrees. Missing values indicate the method is unsuitable for beta-barrel proteins.

We also tested the potential against the recently crystallised proton-gated urea channel HpUreI from *Helicobacter pylori*, a structure consisting of six protomers assembled in a hexameric ring surrounding a central bilayer plug of ordered lipids [32]. Applying the potential and GA to the unaligned structure, it was possible to position the channel such that the lipid vector average, formed by the vectors connecting the terminal carbon atoms in each of the lipid molecules in the cytoplasmic leaflet, was tilted from the z-axis by only 1.54 degrees. We also applied the potential in combination with a slow exhaustive search of all possible orientations, resulting in a lowest energy orientation where the lipid vector average was exactly parallel to the z-axis.

### Assessment of calculated membrane thickness

We compared our estimates of membrane thickness with OPM calculated and experimentally determined values for 12 alpha-helical and beta-barrel targets (Table 3). Experimental values were obtained from site-directed spin labelling studies, cryo-electron microscopy data, X-ray scattering or hydrophobic matching experiments [18]. Calculated values agree well with both experimental values and OPM. Compared to OPM on the targets in our dataset, there is generally good agreement, with an average discrepancy in membrane thickness of 1.8 Å across 125 alpha-helical complexes and 0.9 Å across 37 beta-barrels.

**Table 3:** Calculated membrane thickness for 12 targets where experimentally determined values are available

**Protein**	**PDB**	**MP thickness**	**OPM thickness**	**Experimental thickness**
FepA	1fep	23.00	24.3 ± 1.1	≥23.1
Gramicidin A	1grm	22.25	23.3 ± 4.0	~22
Rhodopsin	1gzm	36.75	32.2 ± 1.5	~30
OmpF	1hxx	23.50	24.2 ± 0.8	~21
Calcium AT Pase	1iwo	29.50	30.8 ± 1.4	~27
BtuB	1nqe	23.50	23.4 ± 1.0	≥20.2
Bacteriorhodopsin	1py6	31.25	24.0 ± 8.0	~32
KcsA channel	1r3j	33.50	34.8 ± 1.2	~34
Photosynthetic reaction centre	1rzh	31.25	31.6 ± 1.4	~30
Cytochrome c oxidase	1v55	30.00	27.8 ± 0.9	~27
Nodium AT Pase	1yce	34.25	37.0 ± 0.5	≥34.5
Mechanosensitive channel	2oar	32.25	36.4 ± 2.2	24

Our potential is 'MP’. Thickness is measured in Angstroms.

### Refinement of alpha-helical transmembrane protein models

Table 4 summarises the performance of the combinatorial refinement algorithm incorporating the membrane potential when tested on 28 models generated by FILM3, showing TM-scores calculated over all helical Cα residues. The TM-score is intended to be a more accurate measure of structural alignment compared to RMSD or GDT. Scores are in the range (0,1], with 1 indicating a perfect match between two structures, scores below 0.20 typically correspond to randomly chosen unrelated proteins, while scores >0.5 are roughly the same fold [33]. Ten different weights were used for the membrane potential term, with a value of 1.6 producing the most consistent results. Compared to models generated using the standard combinatorial refinement procedure (column 2), models generated with the membrane potential (column 3) show an improved TM-score (> = 0.01) in 18 cases, with an average improvement across these 18 targets of 0.05. While increases in TM-score were generally modest, eight targets have TM-score increase of over 0.06, while two are over 0.1 (PDB IDs 2nq2A and 3dhwA). Only three targets have lower TM-scores after refinement with a decrease of 0.03 in the worst case, while seven targets remain unchanged. Across all 28 targets, the average TM-score change is 0.03. We also performed a second round of refinement using MODELLER following refinement using the membrane potential, comparing the resulting models to the final FILM3 models which had also been refined using MODELLER (columns 5–7). Results are similar with 16 targets improved, 6 unchanged and 6 made worse, again by only 0.03 in the worst case, and an average TM-score change is 0.03. These results indicate that different aspects of the structure are refined by the membrane potential and by MODELLER, suggesting that using both in combination should produce the best quality models. Across all Cα residues, performance is slightly less pronounced with 16 targets improved and 3 made worse, with an average TM-score change of 0.02. In terms of the positional accuracy lost by reducing the GA pool size, the maximum observed tilt error with a pool size of 500 was typically double that observed with a pool size of 10000.

**Table 4:** TM-scores of models refined using the membrane potential

	**MP refined**			**MP and MODELLER refined**		
**Target**	**FILM3 recombined**	**Post-refinement**	**Delta TM-score**	**FILM3 recombined**	**Post-refinement**	**Delta TM-score**
1fftC	0.67	0.76	0.08	0.67	0.77	0.10
1gzmA	0.80	0.82	0.02	0.79	0.82	0.03
1ldiA	0.71	0.72	0.02	0.74	0.75	0.00
1pw4A	0.71	0.74	0.03	0.72	0.75	0.03
1xqfA	0.79	0.79	0.00	0.79	0.79	0.00
2abmH	0.78	0.81	0.02	0.80	0.83	0.03
2b2fA	0.72	0.78	0.06	0.73	0.79	0.06
2d2cN	0.67	0.71	0.04	0.69	0.75	0.06
2d57A	0.81	0.79	-0.03	0.82	0.79	-0.02
2f2bA	0.77	0.76	-0.01	0.79	0.79	-0.01
2feeB	0.63	0.70	0.07	0.65	0.71	0.06
2nq2A	0.65	0.76	0.10	0.66	0.74	0.08
2nr9A	0.66	0.73	0.06	0.68	0.75	0.07
2occA	0.81	0.81	0.00	0.83	0.83	0.00
2onkC	0.68	0.66	-0.03	0.68	0.67	-0.01
2q7rA	0.47	0.50	0.03	0.37	0.55	0.18
2qfiA	0.57	0.57	0.00	0.58	0.58	0.00
2r6gG	0.57	0.60	0.04	0.56	0.63	0.07
2witA	0.38	0.38	0.00	0.38	0.39	0.01
2wswA	0.50	0.59	0.09	0.56	0.62	0.06
2ydvA	0.71	0.71	0.00	0.72	0.70	-0.02
2z73A	0.73	0.81	0.08	0.75	0.80	0.06
3b9wA	0.65	0.65	0.00	0.67	0.67	0.00
3dhwA	0.58	0.68	0.10	0.60	0.67	0.07
3mk7A	0.53	0.58	0.05	0.58	0.60	0.01
3mktA	0.73	0.73	0.00	0.73	0.73	0.00
3pjzA	0.78	0.79	0.01	0.80	0.78	-0.02
3qnqA	0.61	0.62	0.01	0.63	0.60	-0.03

Scores were calculated using helical Cα residues only. Columns 2–4 compare models refined using the membrane potential with the recombined FILM3 models. Columns 5–7 compare models refined using the membrane potential and MODELLER with the final FILM3 models, which were also refined using MODELLER.

### Decoy discrimination performance

**Figure 2:**
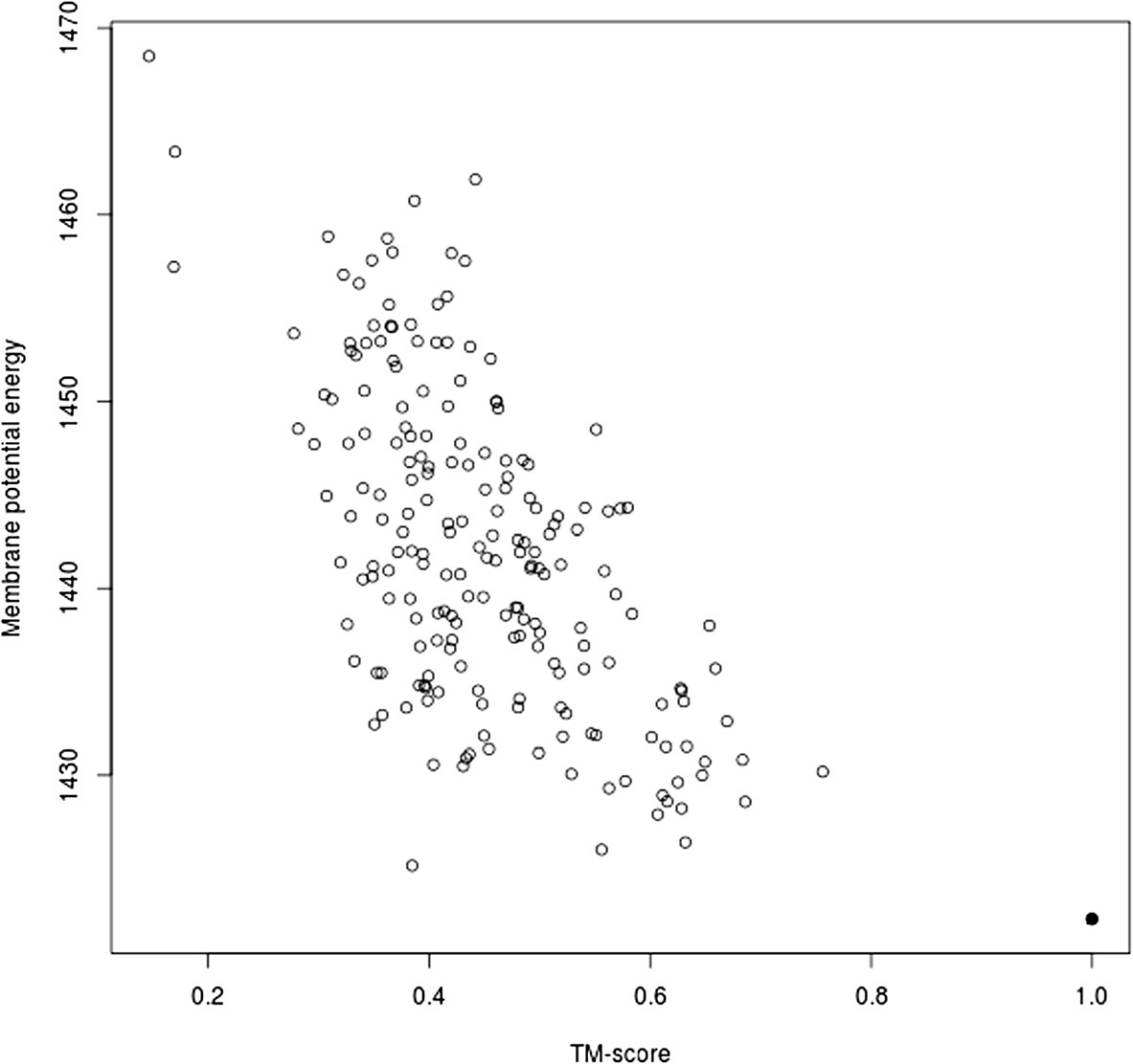
**Scatter plot showing membrane potential energy against TM-score for cyotochrome c oxidase (PDB ID 2occA), Pearson’s r = -0.63.** The model of the native structure is shown as a black square.

Table 5 shows the performance of the membrane potential at discriminating homology models of the 28 FILM3 native structures from the 200 candidate models. Results indicate that the native structure model is correctly identified as the lowest energy structure in 32.1% of cases, while it is amongst the 10 lowest energy structures in 60.7% of cases. The potential is unable to rank two targets effectively, although in both cases they are well positioned in the membrane. Target 2qfiA, the zinc transporter YiiP, is a homodimer held together by four Zn^2+^ ions in its native state, possibly explaining why the potential is unable to reliably identify the monomeric native structure, while 3mktA, the multiple-drug resistance efflux pump, undergoes significant conformational change during transport with the outward-facing form showing high affinity for monovalent cations, suggesting the native form here may not be at its lowest energy state [34,35]. The correlation coefficient between membrane potential energy and TM-score is always negative, with a maximum of -0.63 where the native model is also ranked first (2occA, Figure 2).

**Table 5:** Decoy discrimination results

**Target**	**Min TM-score**	**Max TM-score**	**Pearson’s r**	**Native model rank**
1fftC	0.41	0.68	-0.43	23
1gzmA	0.51	0.69	-0.22	20
1ldiA	0.35	0.71	-0.29	59
1pw4A	0.39	0.69	-0.47	1
1xqfA	0.31	0.71	-0.54	1
2abmH	0.40	0.78	-0.06	2
2b2fA	0.35	0.77	-0.50	1
2d2cN	0.27	0.61	-0.41	28
2d57A	0.41	0.78	-0.38	1
2f2bA	0.38	0.75	-0.34	5
2feeB	0.23	0.63	-0.54	1
2nq2A	0.29	0.68	-0.20	2
2nr9A	0.47	0.67	-0.23	63
2occA	0.15	0.69	-0.63	1
2onkC	0.42	0.68	-0.27	26
2q7rA	0.20	0.50	-0.36	10
2qfiA	0.25	0.52	-0.25	182
2r6gG	0.35	0.62	-0.44	1
2witA	0.19	0.46	-0.49	1
2wswA	0.22	0.55	-0.48	1
2ydvA	0.56	0.73	-0.23	6
2z73A	0.50	0.68	-0.02	93
3b9wA	0.33	0.62	-0.43	2
3dhwA	0.43	0.64	-0.25	2
3mk7A	0.23	0.62	-0.48	8
3mktA	0.46	0.75	-0.01	142
3pjzA	0.32	0.68	-0.44	13
3qnqA	0.24	0.59	-0.17	22

Minimum and maximum TM-scores indicate the range of TM-scores amongst the 200 candidate models per target. PCC is the Pearson’s r correlation coefficient. Native rank is the ranking of the native structure homology model.

## Discussion

In this paper we have developed a knowledge-based membrane potential, calculated from a statistical analysis of transmembrane protein structures, coupled with a genetic and direct search algorithms, and demonstrated its use in positioning proteins in membranes, estimating membrane thickness, refinement of transmembrane protein models and in decoy discrimination. Given the recent increase in the number of high resolution transmembrane protein crystal structures, computational tools which allow proteins to be positioned in membranes are increasingly important as they allows us to study protein-lipid interactions and provide insight into the relationship between sequence, structure and the lipid environment, in a way that isn’t possible using experimental techniques due to the difficulty in crystallising both membrane proteins and lipid molecules.

Compared to other computational approaches such as OPM that are capable of orientating membrane proteins, our method is in extremely good agreement with generally only very small differences in tilt angle, z-coordinate shift and membrane thickness. Although the scarcity of experimental data with which to validate such methods remains an issue, calculated tilt angles are in close agreement with ATR-FTIR spectroscopy determined values and are actually closer to these experimental values than the three other methods tested, while calculate membrane thickness also correlate well with experimental values. However, perhaps the most significant improvement over other methods is the use of GA and direct searches to orientate structures and the consequential speed increase which allows the method to be incorporated into folding or refinement simulations, with up to ~150 orientation calculations per second possible on a single CPU. While our approach provides the option to perform a slower grid search of rotation and translation parameters, both genetic algorithm and direct searches are fast and sufficiently accurate in positioning structures. In a typical case, we can orientate target 4ea3 (278 residues) to within the error of OPM positioning in ~1 second using direct search, under ten seconds using a GA and compared to 231 seconds using a grid search. It is not straightforward to assess the speed of methods such as TMDET and PPM since they run as web servers, although it seems that results are available in ~20 seconds for a typical protein, while Ez-3D takes about 1 second for each 5 residues. The speed of the method, in combination with the freely available source code, should facilitate a wide range of applications for which the other server-based methods are unsuitable. These include the large scale pre-positioning of both alpha-helical and beta-barrel structures into membranes prior to both coarse-grained [16,17] and atomistic molecular dynamics simulations [16,36], for which the computational expense of orientating structures is significant. The method should also be useful for guiding membrane protein design experiments by allowing quantitative predictions to be made regarding the membrane insertion favourability, tilt angle and z-coordinate shift of a sequence, allowing rapid iterative optimisation of stability [24,37-40], while the asymmetric nature of the potential should allow the influence of point mutations on transmembrane topology to be investigated as in the case of the dual topology protein EmrE [41]. Although results obtained using the GA are more accurate, the direct search can be used to obtain a reasonably good orientation but significantly faster. For certain use cases where only an approximate orientation is sufficient (i.e. assessment of topology during a folding simulation), this accuracy/speed trade off may be preferable.

The use of the potential in refinement of alpha-helical transmembrane protein models demonstrates that it is capable of making both significant and consistent contributions to structure prediction. Results from previous CASP refinement experiments indicate that very few groups are capable of making consistent improvements across all targets, and in many cases more harm is done than good [42,43]. Here we have shown that the majority of targets can be improved, by up to 0.1 TM-score units in best cases, with only three targets made worse, and on average by less than 0.03 TM-score units. In addition to directly improving the model, the orientation achieved and the implicit positioning of the membrane provides the foundation for the application of additional membrane-associated terms likely to assist in de novo folding. For example, the positioning of a candidate structure during a folding simulation can be used to determine if the model satisfies its predicted transmembrane topology. A topogenic term can thus be used to score models and therefore encourage them towards adopting the correct topology – an approach equivalent to the use of predicted z-coordinates [44] and likely to be more informative than applying distance constraints from simple linearly extrapolated z-coordinate approximations, which was previously shown to be useful in only 6 out of 28 cases [10], while the application of a lipid exposure potential derived from sequence-based machine learning approaches may also help guide folding to higher resolutions [45]. However, despite the positive contribution towards modelling the transmembrane region, modelling of extra membranous loops regions still requires specific strategies tailored to the physicochemical properties of the membrane-water interface region [46-49]. Future modification of the potential to capture these features may address this issue, while also enabling the positioning of peripheral membrane proteins.

## Conclusions

Overall, we have demonstrated that the potential can be used to accurately position proteins within the membrane, make important contributions to folding simulations and effectively discriminate between native and decoy structures. This approach can be used to gain insights into protein-lipid interactions while assisting in a variety of studies including molecular dynamics, protein design, mutagenesis experiments and transmembrane protein structure prediction.

## Methods

### Membrane potential definition

Alpha-helical and beta barrel membrane potentials were calculated by the statistical analysis of transmembrane protein structures that had been pre-positioned with respect to the bilayer. We used OPM [19,20] to assemble a data set of alpha-helical and beta-barrel proteins, using rotational and translational positions with respect to the membrane as defined by PPM [20]. Chains were homology reduced using the PISCES server [50,51] at the 40% sequence identity level, leaving 183 alpha-helical and 37 beta-barrel chains with a resolution below 3.5 Å. The membrane was modelled as an infinite slab, 48 Å in thickness, divided along the z-axis (perpendicular to the Cartesian plane formed by the membrane surface) into 32 1.5 Å slices – corresponding to the approximate translation per residue in alpha helices - with z = 0 lying at the centre of the membrane, and the cytoplasm in the negative z direction. The frequency of occurrence of each residue’s Cβ (Cα for glycine) atom within a membrane slice was then calculated, adding pseudocounts of one where no residues of a given type were found in a slice, allowing membrane pseudo-energy to be computed for a structure by summing the log likelihood ratios (Equation 1). We also tried alternate formulations based on the inverse Boltzmann equation but in each case they resulted in slightly lower performance [8,24].

(1) $$E_{a}\left(z\right)=-\ln\frac{f_{a}\left(z\right)}{f\left(z\right)}$$

Equation 1. Membrane pseudo-energy for residue *a* at depth *z*, where *f*_*a*_(*z*) is the observed relative frequency of occurrence of amino acid type *a* at depth *z,* and *f*(*z*) is the observed relative frequency of occurrence of all amino acids found at depth *z.*

### Orientation using genetic and direct search algorithms

We used a GA to position structures within the membrane, optimising x and y-axis rotation and z-axis translation such that the membrane potential energy of the structure was minimised. The GA is initialised with a population of 10000 randomly generated individuals. In each generation, the fittest individuals are identified and used as parents for subsequent generations, which are then subject to crossover and mutation operations. Using a GA to efficiently search a large space of possible orientations generally allows an optimal solution to be found relatively quickly, while performance can be further increased as necessary for folding or refinement simulations by reducing the initial population size or limiting the maximum number of energy function calls, at minimal cost to orientation accuracy. However, the final solution may not be the global optimal as GAs can become trapped in local minima, and results can also be inconsistent, even when re-running a GA with the same target or parameters, due to the stochastic nature of the process [52]. We also made use of the Hooke and Jeeves direct search algorithm [53], which is a simple numerical optimisation algorithm that does not require the derivative of the function, thus allowing functions that are not continuous or differentiable to be optimised. The algorithm proceeds by varying one parameter at a time by steps of the same magnitude. When no further increase or decrease in energy is achieved, the step size is modified by a resizing parameter and the process repeated until a termination condition is met. Similarly to GAs, local minima can prevent the optimum solution from being found, particularly where the resizing parameter is set low. We also performed a slow grid search of all rotation and translation parameters, tilted to the nearest degree and translated to the nearest 0.5 Å. While the results of the grid search are always consistent, location of the global energy minimum isn’t guaranteed due to the step size of these parameters.

In order to compare positioning with OPM, targets were first subjected to random rotation about the x and y axes and random translation along the z-axis prior to orientation using the membrane potential and genetic algorithm. Based on OPM topology, the longitudinal axis was then calculated as the vector average of all transmembrane segment vectors, while the mean z-coordinate was calculated using all transmembrane segment boundary residues, and both values compared with OPM. When generating and testing potentials, stringent cross-validation was performed with any structures with greater than 25% sequence identity to the target, or members of the same OPM super family, excluded from the dataset. Potentials were also generated using a range of resolution thresholds in order to assess whether the use of higher resolution structures improved positional accuracy.

### Estimating membrane thickness

Once orientated, we make an estimate of the hydrophobic thickness of the membrane by applying a split potential model of variable thickness. The regions of the potential that encompass the lipid head groups (10 Å ≤ z ≤ 20 Å and -10 Å ≥ z ≥ -20 Å) are translated independently along the Z-axis, with residues in between and outside these regions given the average membrane core and extra-membranous propensity scores for that residue type, respectively. The effects of applying these translations is to sample the pseudo-energy landscape as a function of variable lipid tail lengths. By identifying the translations for each of the two regions that in combination result in the lowest pseudo-energy, membrane thickness can be estimated by measuring the distance between them, based on a standard hydrophobic thickness of 30 Å.

### Membrane protein structure refinement

We tested the contribution of the membrane potential to structure refinement by incorporating it as a second energy term in the combinatorial refinement algorithm we have described previously in our *de novo* modelling method FILM3 [10]. In FILM3, an ensemble of 200 models was generated for each of 28 alpha-helical membrane protein targets using the standard FILM/FRAGFOLD approach [8,28] though with the energy function replaced by a distance constraint function based solely on residue contacts predicted by PSICOV [54], and Replica Exchange Monte Carlo in place of simulated annealing for the conformational search using structural fragments. The combinatorial refinement protocol involves superposing the 100 lowest energy models onto the lowest energy model, before selecting random fragments from each model and transferring these onto the equivalent chain segment in the lowest energy structure. Where a lower energy model is produced, this is retained and the greedy search procedure repeated until no further improvement in energy is observed. In most cases, this procedure allowed a final model to be generated with an energy value lower than any of the 200 candidate structures. Here, we generated ensembles for each of the 28 targets but using the recombined structures as the lowest energy model onto which the 100 lowest energy models were superposed, therefore minimising the possibility that any subsequent improvement in model quality could be attributed to further satisfaction of predicted contacts. The membrane potential term was weighted and combined with the distance constraint term and a total of 5 million fragment swaps carried out, with the genetic algorithm population size reduced from 10000 to 500 in order to improve compute time (Equation 2).

(2) $$E_{\mathrm{Total}}=E_{\mathrm{Contact}}+w.E_{\mathrm{Membrane}}$$

Equation 2. Total pseudo-energy for a structure, E_Contact_ is the FILM3 distance constraint term [10] and w is an adjustable weight in the range 0.1 to 2.0.

### Decoy discrimination

Finally, we examined the ability of the potential to discriminate near native from decoy alpha-helical membrane protein targets, again using the 200 models generated for each of 28 targets generated by FILM3. Since our knowledge-based potential is “trained” using experimentally determined structures, it may well capture the intrinsic properties of native conformations well but often such potentials fail to evaluate the quality of near-native and misfolded conformations appropriately [55]. Rather than using the experimental structures, we therefore built homology models with MODELLER [56] using the native crystal structures as templates. We then evaluated the performance of the potential at discriminating these homology models from the 200 decoys, assessing the frequency with which lowest energy conformation having the highest TM-score [33], the frequency with which the lowest energy conformation was amongst the top 10 TM-scores and the correlation coefficient between membrane potential energy and TM-score.

### Availability of supporting data

Source code is available under an open source license from the URL below. In order to compile and run, the g++ compiler and Boost C++ libraries are required.

http://bioinf.cs.ucl.ac.uk/downloads/memembed/

## Competing interests

The authors declare that they have no competing interests.

## Authors’ contributions

TN designed and performed research. Both authors drafted the manuscript, revised it critically and read and approved the final version.
